# Bacterial Necromass Is Rapidly Metabolized by Heterotrophic Bacteria and Supports Multiple Trophic Levels of the Groundwater Microbiome

**DOI:** 10.1128/spectrum.00437-22

**Published:** 2022-06-14

**Authors:** Patricia Geesink, Martin Taubert, Nico Jehmlich, Martin von Bergen, Kirsten Küsel

**Affiliations:** a Aquatic Geomicrobiology, Institute of Biodiversity, Friedrich Schiller University, Jena, Germany; b Laboratory of Microbiology, Wageningen University and Research, Wageningen, the Netherlands; c Department of Molecular Systems Biology, Helmholtz Center for Environmental Research-UFZ GmbH, Leipzig, Germany; d Institute of Biochemistry, Pharmacy, and Psychology, University of Leipzig, Leipzig, Germany; e German Centre for Integrative Biodiversity Research (iDiv) Halle-Jena-Leipzig, Leipzig, Germany; University of Massachusetts Amherst

**Keywords:** groundwater, necromass, surface input, stable isotope probing, metaproteomics, subsurface, AquaDiva, groundwater

## Abstract

Pristine groundwater is a highly stable environment with microbes adapted to dark, oligotrophic conditions. Input events like heavy rainfalls can introduce the excess particulate organic matter, including surface-derived microorganisms, thereby disturbing the groundwater microbiome. Some surface-derived bacteria will not survive this translocation, leading to an input of necromass to the groundwater. Here, we investigated the effects of necromass addition to the microbial community in fractured bedrock groundwater, using groundwater mesocosms as model systems. We followed the uptake of ^13^C-labeled necromass by the bacterial and eukaryotic groundwater community quantitatively and over time using a complementary protein-stable and DNA-stable isotope probing approach. Necromass was rapidly depleted in the mesocosms within 4 days, accompanied by a strong decrease in Shannon diversity and a 10-fold increase in bacterial 16S rRNA gene copy numbers. Species of *Flavobacterium*, *Massilia*, *Rheinheimera*, *Rhodoferax*, and *Undibacterium* dominated the microbial community within 2 days and were identified as key players in necromass degradation, based on a ^13^C incorporation of >90% in their peptides. Their proteomes comprised various proteins for uptake and transport functions and amino acid metabolization. After 4 and 8 days, the autotrophic and mixotrophic taxa *Nitrosomonas*, *Limnohabitans*, *Paucibacter*, and *Acidovorax* increased in abundance with a ^13^C incorporation between 0.5% and 23%. Likewise, eukaryotes assimilated necromass-derived carbon either directly or indirectly. Our data point toward a fast and exclusive uptake of labeled necromass by a few specialists followed by a concerted action of groundwater microorganisms, including autotrophs presumably fueled by released, reduced nitrogen and sulfur compounds generated during necromass degradation.

**IMPORTANCE** Subsurface microbiomes provide essential ecosystem services, like the generation of drinking water. These ecosystems are devoid of light-driven primary production, and microbial life is adapted to the resulting oligotrophic conditions. Modern groundwater is most vulnerable to anthropogenic and climatic impacts. Heavy rainfalls, which will increase with climate change, can result in high surface inputs into shallow aquifers by percolation or lateral flow. These inputs include terrestrial organic matter and surface-derived microbes that are not all capable to flourish in aquatic subsurface habitats. Here, we investigated the response of groundwater mesocosms to the addition of bacterial necromass, simulating event-driven surface input. We found that the groundwater microbiome responds with a rapid bloom of only a few primary degraders, followed by the activation of typical groundwater autotrophs and mixotrophs, as well as eukaryotes. Our results suggest that this multiphase strategy is essential to maintain the balance of the groundwater microbiome to provide ecosystem services.

## INTRODUCTION

Life in the subsurface is shaped by the lack of photosynthesis-driven primary production, leading to limited availability of organic carbon (OC) and a dependency on chemolithoautotrophy in subsurface environments like groundwater (1–3). Metagenomics-based studies have pointed out that indeed chemolithoautotrophic microorganisms are key players within groundwater (4–7) that can provide the scarce OC to other members of the microbial community and thus fuel complex food webs (2, 8, 9). However, the connectivity to the surface also evokes input of OC via seepage in surface-near groundwaters, especially during events like heavy rainfalls or snowmelts (10–12). The majority of OC formed at the surface is photosynthetically derived carbon (i.e., plant material, root exudates). This event-driven inflow of large amounts of dissolved and particulate organic matter fuels the groundwater microbiome with impacts on its diversity (10, 13, 14).

Soil seepage can contain high numbers of bacterial cells, in abundances one to 2 orders of magnitude higher than groundwater (11). Microbial transformation of OC in soils gradually shifts plant-derived OC signatures to microbially derived compounds (15). During heavy rainfall or snowmelt events, seepage-derived microbial cells will bypass surface recycling of biomass due to rapid transport into the groundwater (10, 11, 13–18). While a fraction of the introduced microbial biomass will be able to thrive in the oligotrophic groundwater leading to an input-driven succession of the bacterial community (11, 16), most translocated cells presumably die and form a pool of dead microbial biomass (necromass), another OC source for native groundwater microorganisms.

Microbial necromass consists mainly of proteins (50%), RNA (20%), and small molecules (13%), providing amino acids and other central metabolites that can be assimilated by microorganisms. The ability to use necromass as a source of OC has been shown in different environments like marine sediments, soils, and compost (12, 19–24). While necromass-derived compounds should be accessible to a wide range of microorganisms, studies from marine and terrestrial environments have shown that only a few taxa are involved in the degradation of necromass in their respective habitat (20, 21). In addition, members of the candidate phyla radiation (CPR), a clade of bacteria that lack critical metabolic pathways (17, 25, 26), have been hypothesized to use necromass as a source of OC and metabolites that they cannot synthesize themselves (27–29). However, key players in necromass recycling and its impact on the complex communities in groundwater remain elusive.

Here, we investigated fractured bedrock groundwater obtained from the Hainich Critical Zone Exploratory (CZE) (30) to study the implications of necromass for complex microbial communities in surface-near groundwater. We hypothesize that necromass input to groundwater results in a drastic change in the microbial community. Furthermore, we assume that this disturbance leads to the distinct response of specific taxa, followed by dissemination of the assimilated carbon through the groundwater community, including members of the CPR. By adding necromass of a groundwater isolate to groundwater mesocosms, we followed the uptake of ^13^C on DNA- and protein-level by a complementary stable isotope probing (SIP) approach (31, 32). Besides being able to detect microorganisms that take up necromass, SIP-metaproteomics allowed us to identify the most abundant proteins and link them to a potential lifestyle of the respective organism within our incubations. To elucidate the potential implications of necromass recycling across trophic levels, we investigated both bacterial and eukaryotic community members. Our results revealed microbial key players that rapidly respond to event-driven necromass input in groundwater and demonstrated a concerted action of groundwater organisms from different trophic levels.

## RESULTS

### Rapid depletion of Pseudomonas necromass.

**FIG 1:**
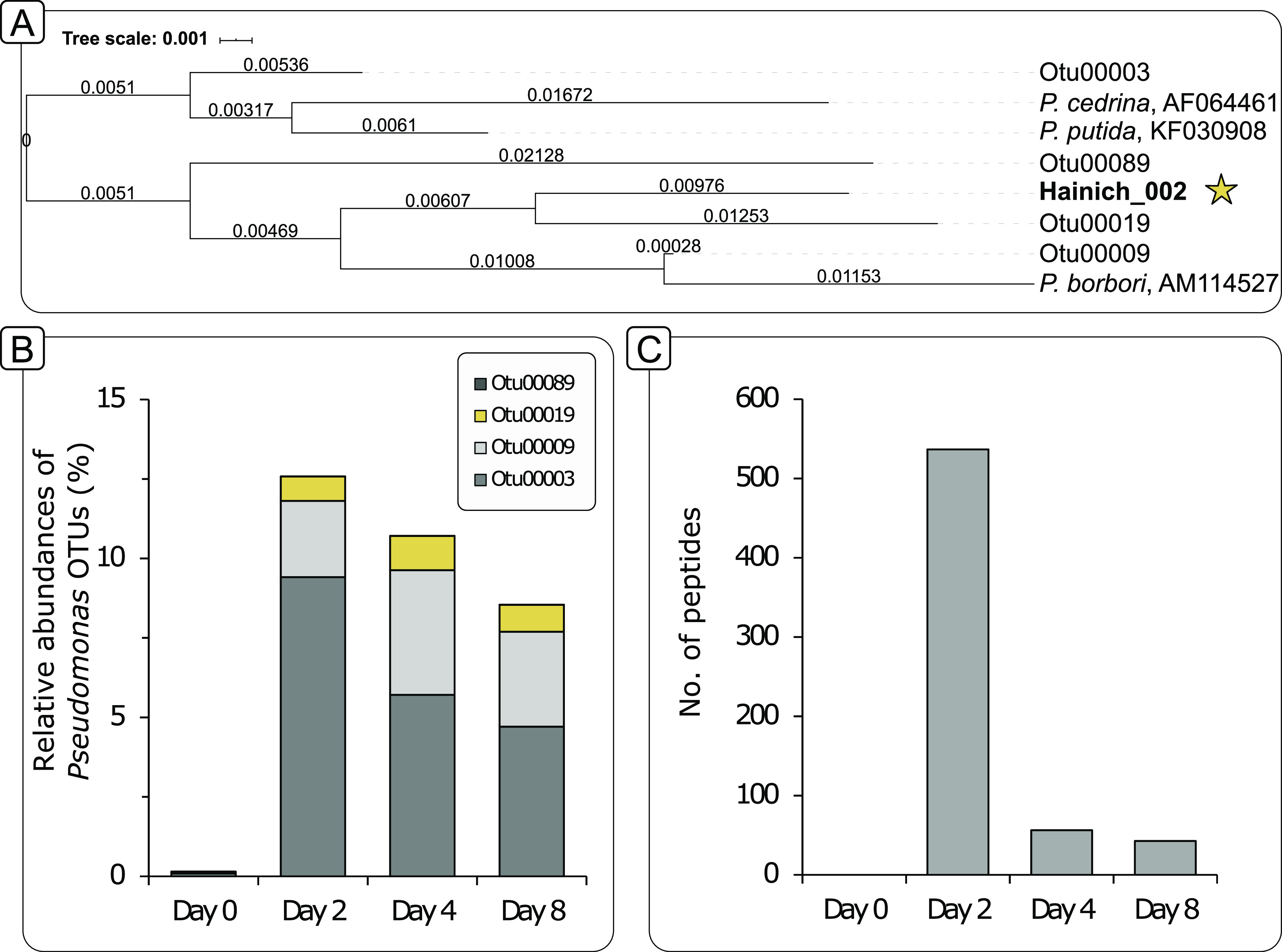
Abundances of Pseudomonas on DNA and peptide level. (A) Phylogenetic tree based on 16S rRNA gene sequences of the most abundant Pseudomonas OTUs as well the isolate Hainich_002 that was used for necromass generation (star symbol). The tree was calculated using the arb neighbor-joining method (1000 bootstraps) within arb (81, 82). Closely related Pseudomonas species were added as references. (B) Relative abundances of Pseudomonas-related OTUs increase after necromass was added on day 0 and then progressively decrease throughout the incubation. Likely, also members of the genus Pseudomonas partake in the initial degradation of Pseudomonas-derived necromass. OTU00019, the most closely related OTU to the isolate Hainich_002 is highlighted in yellow. (C) Number of peptides associated with the genus Pseudomonas within the original groundwater at day 0, as well as the ^12^C labeled incubations over time. Unlike at the DNA level, most Pseudomonas-related peptides presumably stem from the added necromass.

Necromass-derived peptides were rapidly depleted in the groundwater mesocosms during incubation, dropping to 56.06% ± 2.15% relative abundance on day 2 of incubation and further to only 2.41% ± 0.25% on day 4 (Fig. 1C). This explicit decrease suggested a rapid degradation of necromass by the groundwater microbial community within the first 2 days of the incubation. These peptides featured a high ^13^C relative isotope abundance (RIA) of 98% (average across all peptides at day 2, Fig. S1) in mesocosms supplemented with ^13^C-labeled necromass and were exclusively affiliated with Pseudomonas, clearly identifying them as necromass-derived.

Pseudomonas operational taxonomic units (OTUs) only decreased marginally in relative abundance over incubation time. Sequence analysis revealed that these OTUs were distinct from Pseudomonas isolate Hainich_002 used for the generation of necromass (Fig. 1A). The most abundant Pseudomonas OTUs showed only 95% to 98% sequence identity to this isolate. The OTU with the highest identity (OTU00019; 97.8%) constituted less than 2% of the microbial community (Fig. 1B). Because no amplicon could be generated from the original necromass suspension, it can be assumed that necromass was not detected at the DNA level in the mesocosms, allowing less-biased profiling of the bacterial community structure.

### Specific bacterial taxa respond to the addition of necromass.

**FIG 2:**
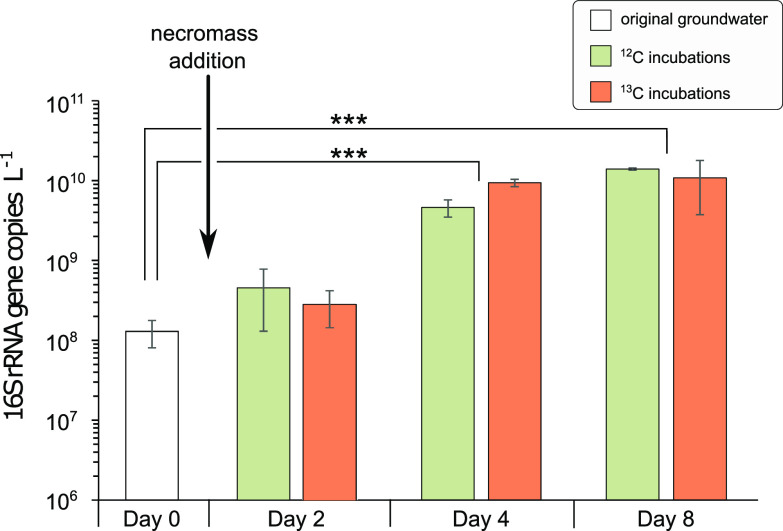
Bacterial abundances in groundwater and mesocosms supplemented with necromass. Average 16S rRNA gene copy numbers within the original groundwater (white), as well as within the incubations on days 2, 4, and 8 after necromass addition in the ^12^C (green) and ^13^C (orange) mesocosms. Error bars show the standard deviation of three samples. *P* values were derived from Student's *t* test and indicated by asterisks (*, *P* < 0.05; **, *P* < 0.01; ***, *P* < 0.001).

**FIG 3:**
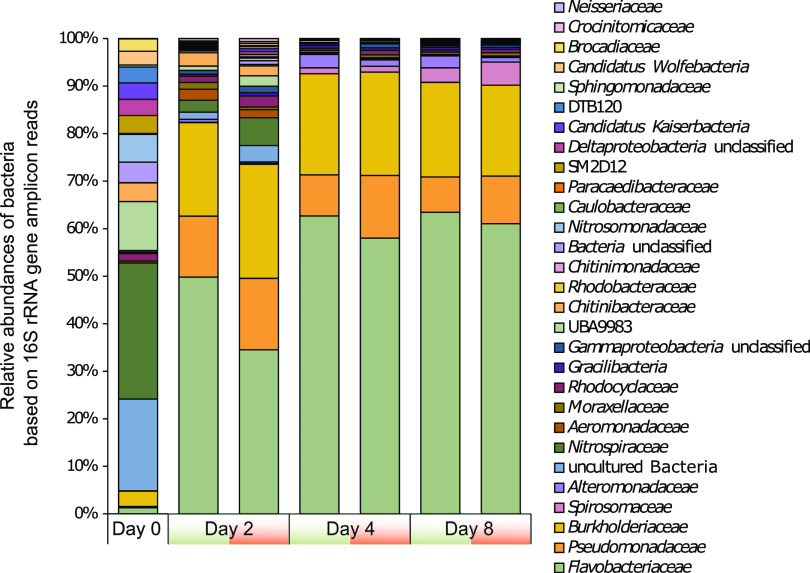
Phylogenetic profiles based on relative abundances of bacterial 16S rRNA gene amplicons. Average relative abundances of bacterial families in the original groundwater, as well as the ^12^C (green) and ^13^C (orange) mesocosms over the 8 days of incubation. Shannon diversity indices are given as average values per time point.

Bacterial cell abundances (based on 16S rRNA gene copy numbers) increased 1 order of magnitude within 4 days after the addition of necromass, from 1.3 × 10^8^ ± 4.9 × 10^7^ to 7.0 × 10^9^ ± 2.6 × 10^9^ gene copies per liter of groundwater, indicating bacterial growth within the mesocosms (Fig. 2). This increase was significant after four and 8 days compared to unamended groundwater (*P*_Day4_ = 0.0001 and *P*_Day8_ = 0.00003, Student's *t* test). Shannon diversity decreased significantly from the original groundwater to day 2 of the incubation, from 5.67 ± 0.09 to 3.82 ± 0.38, respectively (*P* = 4 × 10^−5^, Student's *t* test) and continued to decrease thereafter (Fig. 3 and Table S2).

The original groundwater community was dominated by *Nitrospiraceae* and families belonging to the candidate phyla radiation (CPR) superphylum (candidate division UBA9983, *Cand.* Kaiserbacteria, *Cand.* Wolfebacteria, *Cand.* Magasanikbacteria, *Cand.* Parcubacteria, *Cand.* Gracilibacteria, Fig. 3). After 2 days of incubation, combined relative abundances of *Flavobacteriaceae*, *Burkholderiaceae*, and *Pseudomonadaceae* made up 68% of the bacterial community. *Spirosomaceae* and *Alteromonadaceae* increased in relative abundance at the later time points, reaching 5.7% on day 8 (Fig. 3). While only comprising 2.5% of the original groundwater community, these families remained dominant in the mesocosms until the end of the incubation. In contrast, *Nitrospiraceae*, which made up 14.2% of the original groundwater community, decreased to 3.6% after 2 days and are not detected at the later time points.

### Heterotrophic bacteria rapidly degrade necromass.

**FIG 4:**
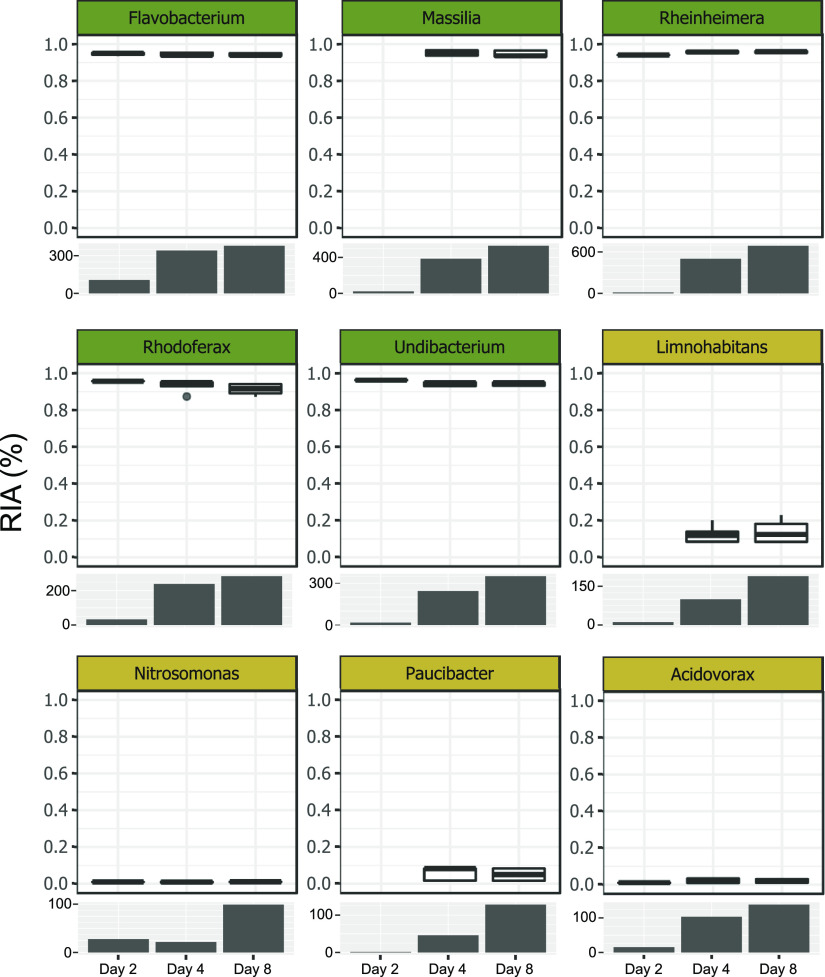
Relative isotopic abundances (RIA) and the number of peptides in the most abundant genera. Boxplots show the uptake of labeled carbon by potential heterotrophic (dark green), autotrophic (yellow), and mixotrophic (blue) bacteria in the ^13^C incubations. The corresponding bar charts represent the numbers of identified peptides in the ^12^C incubations for the respective genus.

To follow the uptake of necromass-derived carbon in the microbial community, we quantified the ^13^C relative isotope abundances (RIAs) in peptides of the most abundant genera using SIP-metaproteomics. The uptake of necromass by heterotrophic members of the groundwater community should result in a high ^13^C-incorporation into the biomass of these bacteria. We observed ^13^C RIAs of 93% to 96% in peptides of the genera *Flavobacterium*, *Massilia*, *Rheinheimera*, *Rhodoferax*, and *Undibacterium* already after 2 days of incubation, pointing toward a very fast and exclusive uptake of labeled necromass (Fig. 4). Simultaneously, a rapid increase in the number of peptides matched to these genera was observed, confirming the detected increase in abundance at the 16S rRNA gene level. The increase in biomass coincided with the depletion of necromass, suggesting the rapid growth to be coupled with necromass utilization.

**FIG 5:**
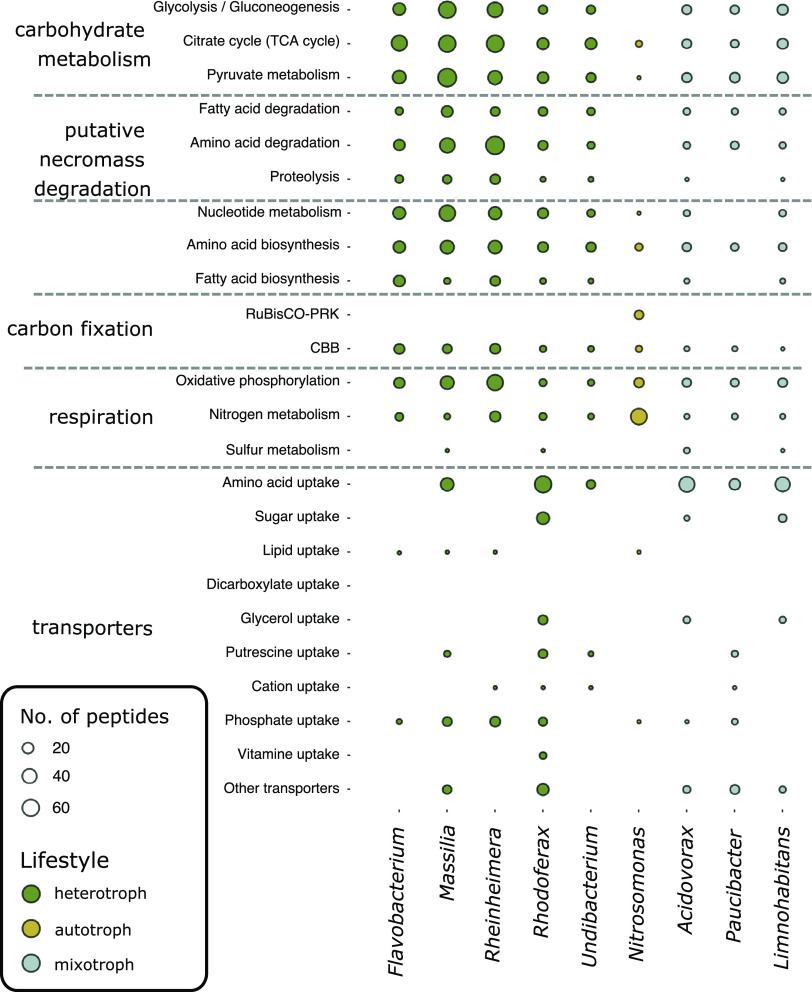
Functional classification (KEGG orthology) of identified peptides from the most abundant genera. Heterotrophic genera (dark green) show high abundances of peptides associated with the degradation and transport of amino acids as well as the other transporting mechanisms potentially involved in the uptake and breakdown of necromass. Potentially autotrophic (yellow) and mixotrophic (blue) bacteria show a less distinct pattern. For *Nitrosomonas* peptides from the key enzyme of carbon fixation via the CBB cycle, as well as enzymes participating in nitrogen metabolism were identified. Circle sizes indicate the number of identified peptides.

The proteomes of these heterotrophic genera exhibited functionalities ideally suited for a lifestyle dependent on necromass. Various uptake and transport-related proteins for the import of necromass, including amino acid, lipid, sugar, and vitamin transporters, as well as many unclassified transporters were detected (Fig. 5 and Table S3). Proteins associated with proteolysis as well as the degradation of amino acids and fatty acids, targeting major components of necromass, were also identified. Further proteins present were involved in central carbohydrate metabolism, the tricarboxylic acid cycle (TCA) cycle, respiration, and cell division, ensuring rapid biomass biosynthesis from necromass and rapid growth (Table S3).

### Diversified secondary response to a bloom of necromass degraders.

Unlike the fast-responding necromass degraders, other bacterial genera showed a slower response in the groundwater mesocosms and proliferated mainly after four to 8 days (based on both 16S rRNA gene and peptide abundances). These genera, including *Nitrosomonas*, *Acidovorax*, *Limnohabitans*, and *Paucibacte*r, did not feature the high ^13^C RIA expected for growth on necromass and only had between 0.5 and 23% ^13^C incorporated in their peptides (Fig. 4). This hinted at the primary use of an unlabeled carbon source for their growth. Their proteomic makeup suggested different lifestyles: *Nitrosomonas* produced enzymes of the Calvin-Benson-Bassham (CBB) cycle, including ribulose-bisphosphate carboxylase/oxygenase (RuBisCO) (Fig. 5). No enzymes involved in proteolysis, amino acid, or fatty acid degradation were identified from this genus, and functionalities for OC uptake were highly restricted. Ammonia monooxygenase and nitrite reductase were present, suggesting nitrification by *Nitrosomonas*. The low ^13^C incorporation (0.99% ± 0.15%) clearly showed that *Nitrosomonas* did not obtain carbon from necromass, and the proteomic makeup indicated a chemolithoautotrophic lifestyle instead.

The proteomes of the genera *Acidovorax*, *Limnohabitans*, and *Paucibacter* were more similar to those of the fast-responding heterotrophs, featuring functionalities for OC uptake, proteolysis, as well as amino acid and fatty acid breakdown (Fig. 5). Individual proteins involved in nitrogen and sulfur metabolism were identified, such as nitric oxide reductase (*Acidovorax*) and SoxAX cytochromes (*Limnohabitans*), respectively (Table S3). Although these organisms have partly been attributed to the ability to fix CO_2_ via the CBB cycle (33, 34), no RuBisCO was detected. The ^13^C incorporation in the peptides of these genera varied between 1.99% ± 1.18% ^13^C (*Acidovorax*) and 13.24% ± 4.92% ^13^C (*Limnohabitans*) (Fig. 4), indicating limited assimilation of necromass-derived carbon.

To confirm the results obtained and to additionally determine the ^13^C incorporation in microorganisms not covered by the SIP-metaproteomic approach, we performed DNA-SIP with samples from the mesocosms incubated for 8 days. Besides the heterotrophic bacterial genera *Flavobacterium*, *Massilia*, *Rheinheimera*, *Rhodoferax*, and *Undibacterium* identified to take up necromass-derived ^13^C using protein-SIP, DNA-SIP revealed a significant enrichment of DNA from OTUs belonging to the genera *Pseudarcicella*, *Iodobacter*, *Chitinimonas*, *Perlucidibaca*, and *Aquabacterium* in the heavy DNA fraction of the ^13^C incubations (Fig. S2), indicating necromass-derived carbon assimilation by these genera. Furthermore, one OTU belonging to the CPR phylum *Cand.* Gracilibacteria was enriched in the ^13^C heavy DNA fraction (Fig. S2).

### Groundwater eukaryotes incorporate necromass-derived carbon.

**FIG 6:**
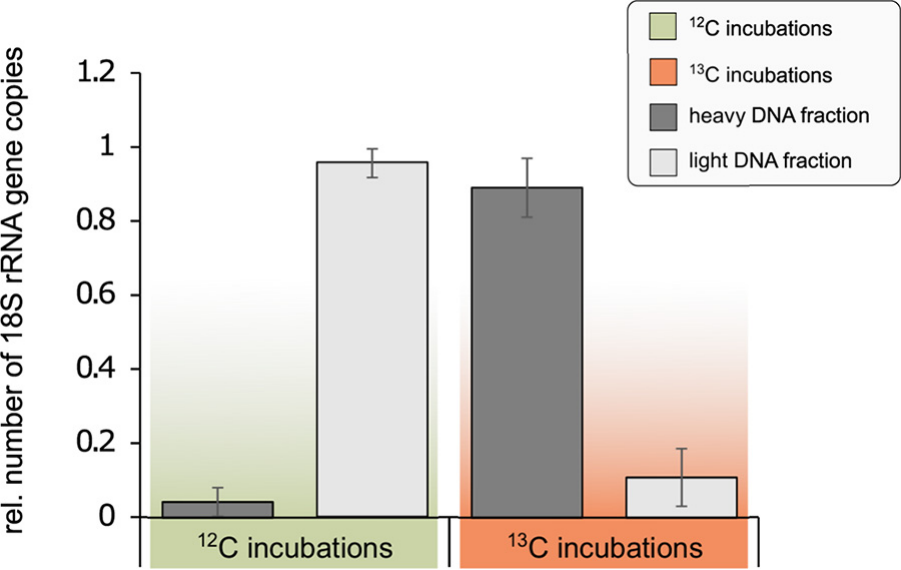
Evidence of necromass uptake by Eukaryotes via DNA-SIP. Relative abundances of eukaryotic 18S rRNA genes in the heavy and light DNA fractions after 8 days of incubation show a significant shift toward the heavy DNA fractions in the incubations with ^13^C-labeled necromass (*P* < 0.001; Fisher’s exact test; Table S5), indicating that eukaryotes have been taking up necromass derived ^13^C during the experiment.

We also observed a strong enrichment of eukaryotic DNA in the ^13^C heavy DNA fractions, based on qPCR targeting 18S rRNA genes. In the ^13^C labeled incubations, 89.13% ± 7.94% of the 18S rRNA gene copies were found in the heavy DNA fraction, compared to 4.24% ± 3.88% in the unlabeled control incubations (Fig. 6 and Table S5). This strong shift from light to heavy DNA fraction indicated assimilation of ^13^C derived from necromass by the eukaryotic community.

## DISCUSSION

**FIG 7:**
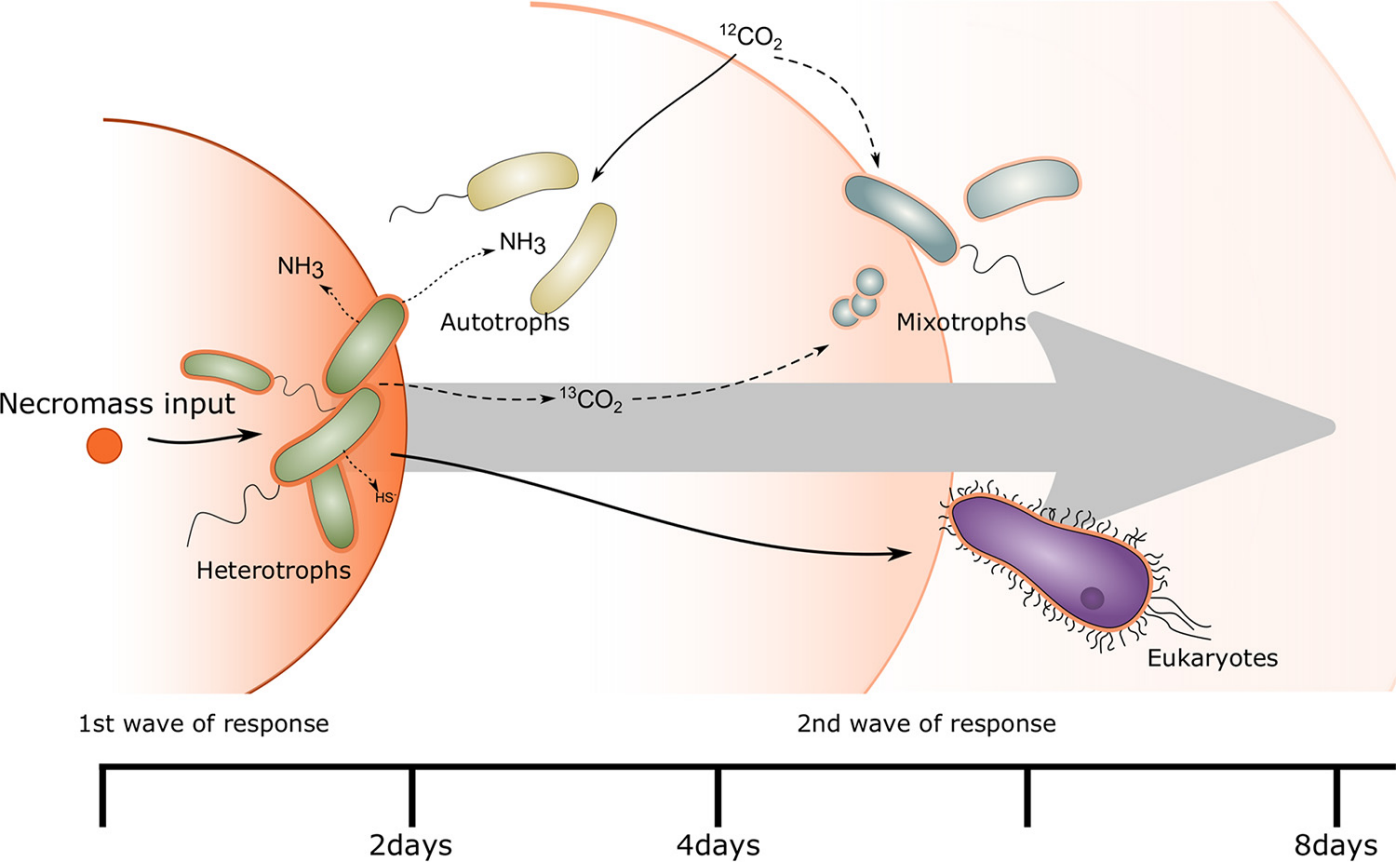
Conceptual view of the response of the groundwater microbiome to a disturbance by large inputs of necromass derived carbon. ^13^C labeled necromass (orange) is taken up by heterotrophic (green) members of the community that can rapidly thrive on the added OC. By metabolizing, e.g., amino acids contained in necromass, excess nitrogen, and sulfur compounds are being released and can subsequently stimulate the growth of autotrophic (yellow) and mixotrophic (blue) bacteria. Simultaneously, eukaryotes (purple) are taking up necromass-derived ^13^C by feeding on the heterotrophic community members or necromass directly.

Microbial communities of surface-near groundwater are not stable over time and exhibit nonlinear dissimilarity patterns corresponding to periods of groundwater recharge (16). Groundwater recharge events represent successive disturbances, driving the temporal variation through their impact on microbial diversity and abundance, including the introduction of surface-derived taxa. Not all these taxa will be able to thrive under groundwater conditions. Their necromass may lead to a pronounced shift in the community composition by promoting the growth of heterotrophs, which will further sustain subsurface communities. The addition of necromass to the groundwater mesocosms led to a rapid response of the groundwater microbiome (Fig. 7). Already within 2 days, the community had changed drastically, with *Flavobacterium*, *Massilia*, *Rheinheimera*, *Rhodoferax*, and *Undibacterium* together accounting for 49% to 63% of the total community. Based on their high ^13^C RIA, these primary degraders exclusively utilized carbon derived from the added necromass and had already consumed more than 90% of it after 4 days. This confirms our hypothesis that only a few species of the groundwater community take over a major part of necromass disposal, leading to a drastic change in the community structure because it has been previously observed in soil and marine microbial communities (24, 35, 36).

Apart from the dominant first responders, further heterotrophic bacteria were found to become labeled. The detected ^13^C incorporation in *Cand.* Gracilibacteria showed that also members of the CPR, which are highly abundant in groundwater (11, 18, 37), can assimilate necromass-derived carbon, as previously suggested (28, 38). Genomic insights suggest a dependency on *Cand.* Gracilibacteria on the uptake of citrate, malate, and amino acids from external sources, due to the lack of glycolysis, the pentose-phosphate and Entner Doudoroff pathways (38). These important biomolecules might be provided through necromass yet cannot sufficiently be used to generate enough energy for fast replication. This likely prevents members of the CPR from competing with fast responding heterotrophs and from playing a major role in necromass disposal. Instead of preferentially responding to event-based inputs of necromass, members of the CPR are thus rather persistent members of the groundwater microbiome (39).

Subsequently, between four and 8 days of incubation, other microbial taxa like *Paucibacter*, *Limnohabitans*, *Acidovorax*, and *Nitrosomonas* increased in abundance, representing a secondary response. These organisms did not rely exclusively on the utilization of necromass-derived carbon but employed other lifestyles according to their ^13^C RIAs. Bacteria of the genus *Nitrosomonas* are well known as chemolithoautotrophic nitrifiers (4, 40). Necromass degradation during heterotrophic growth is accompanied by a loss of carbon via oxidation to CO_2_, and at the same time, release of ammonium from the catabolism of nitrogenous molecules. The obtained proteomic data underpins that *Nitrosomonas* likely oxidized this ammonium in the mesocosms for autotrophic growth. This is in accordance with the low ^13^C RIA observed in these organisms. The groundwater in the mesocosms typically contains around 400 mg/liter bicarbonate (≙ 84 mg ^12^C liter^−1^) (41), so even complete oxidation of the added necromass (0.5 mg ^13^C liter^−1^) and subsequent fixation of all the released CO_2_ would only lead to a maximal ^13^C RIA of 0.6% in the bicarbonate pool available to autotrophs.

The ^13^C RIA of *Acidovorax*, *Limnohabitans*, and *Paucibacter* suggested the limited uptake of carbon derived from necromass. Most of the carbon assimilated by these organisms, however, came from an unlabeled carbon source. This mix of necromass and unlabeled carbon might be the result of a mixotrophic lifestyle, given the described ability of these genera to fix CO_2_ (33, 34), even though proteomic evidence is missing. Also, a purely heterotrophic lifestyle, based on organic carbon from the original groundwater, or organic carbon produced by chemolithoautotrophy, is possible. The delayed response of these genera suggests an indirect role in necromass disposal, potentially by removing compounds not accessible to the primary degraders or compounds released during this initial degradation of necromass.

Furthermore, we observed a transfer of ^13^C into eukaryotic biomass. Protozoa can directly feed on particulate organic matter (42), potentially, including necromass. Thereby, the added necromass potentially serves as a direct food source for groundwater eukaryotes. Additionally, bacteria that partake in the initial degradation of necromass might accumulate around clumps of necromass, and thus lower trophic levels of protozoa which are known to feed on groundwater bacteria (2, 8, 43) can graze on the available bacterial biomass. The pronounced density shift of eukaryotic DNA toward the heavy fraction indicates the uptake of ^13^C, and thus the necromass directly or bacteria that belonged to the primary response. In the long run, predation could in turn lead to a reduction in the abundance of bacterial necromass degraders.

The heterotrophic microbes responsible for the first wave of necromass degradation are typically present in low abundance in groundwater of the Hainich CZE (11, 16, 39). *Rhodoferax* (Burkholderiaceae) was reported as part of the core groundwater microbiome present throughout the transect (39). These organisms might be able to respond to event-driven necromass input *in situ*, explaining in part fluctuations in the groundwater community observed between the recharge and discharge phase (16). Such heterotrophic Proteobacteria and Bacteroidetes are preferentially mobilized from soils during groundwater recharge (11, 13). An input event might thus not just introduce necromass, but also specialists for its disposal into the groundwater. This suggests that irregular input events can play an important role to ensure the upkeep of the necromass degradation potential in groundwater.

Autotrophs like *Nitrosomonas* observed during the secondary response were identified as key players in the nitrogen cycle within groundwater from the Hainich CZE (4). Sulfur oxidizers are abundant in the groundwater and exhibit a highly flexible, mixotrophic lifestyle, and, hence, play an important role in the recycling of organic carbon (44–46). These autotrophs and mixotrophs are part of the core microbiome in the undisturbed groundwater of the Hainich CZE (39). Surface-derived inorganic nitrogen compounds and rock-derived inorganic sulfur compounds might be essential for their growth (47–50). Our study demonstrates that autotrophic microorganisms in the groundwater mesocosm can as well benefit from sulfur and nitrogen compounds generated during necromass degradation. Hence their activity might contribute to necromass disposal and restoration of the native groundwater microbiome after a disturbance.

Eukaryotes like protozoans and metazoans, which are known to feed on bacteria, form the top of the groundwater food web in the Hainich CZE (43, 51). Their abundance and diversity were shown to correlate positively with the abundance of chemolithoautotrophic primary producers (8). Our results demonstrate that following blooms of necromass-degraders, eukaryotes incorporated necromass-derived OC by feeding on the labeled heterotrophic bacteria or necromass directly. Eukaryotes thus benefit indirectly from available necromass following input events. By rapid grazing on locally highly abundant necromass degraders, they could potentially contribute to the recovery of the native state of the groundwater microbiome. The concerted action of heterotrophs, mixotrophs, autotrophs, and eukaryotes not only contributes to the rapid metabolization of necromass-derived OC but potentially also supports the long-term stability of the groundwater microbiome following disturbances caused by surface inputs.

### Conclusion.

Oligotrophic groundwater ecosystems feature highly stable conditions and a microbiome characterized by adaptation to the limited availability of OC and by its high potential for chemolithoautotrophic primary production. Triggered by events like heavy rainfalls, the input of OC in the form of necromass during recharge creates disturbances in the groundwater microbiome. Employing a mesocosm model system, our study shows how the groundwater microbiome uses a multiphase strategy to respond to this disturbance. In the primary phase, a few necromass degraders, some of which originate from the soil itself, rapidly dispose of the majority of the necromass and proliferate greatly in the process. Subsequently, specialized mixotrophs and autotrophs, which also occur in the native groundwater community, remove remaining constituents and waste products, and eukaryotes exert top-down control to return the microbiome to its native state. Necromass degraders persist in low abundance in the groundwater community, and successive disturbances could help to sustain them to preserve the ecosystem function they exert in the groundwater microbiome. The rapid and concerted response over multiple trophic levels might be one of the processes explaining the stability of the groundwater microbiome.

## MATERIALS AND METHODS

### Labeling and generation of necromass.

A bacterial isolate (Pseudomonas sp. [52]), from the Hainich CZE (30) was grown on S2P solid medium (52) for 1 day at room temperature as a preculture. Subsequently, the bacterium was transferred into a minimal medium (0.4 g/liter K_2_HPO_4_, 1.6 g/liter MgSO_4_ × 7H_2_O, 0.8 g/liter NH_4_Cl, 25 mM HEPES, 5 mL trace element solution “T” [53]) containing either 0.9 g/liter ^12^C-labeled or ^13^C-labeled glucose (Sigma-Aldrich) as a carbon source to generate unlabeled and ^13^C-labeled bacterial biomass. Cultures were incubated in sterile 50 mL centrifuge tubes (Greiner bio-one) at room temperature with mild shaking for 3 days. Subsequently, all cultures were centrifuged at 10,000 × *g* for 10 min, the cell pellets were washed in 0.1% NaCl (wt/vol) solution and combined in serum bottles under sterile conditions. Cell numbers in both solutions (labeled and unlabeled) were determined using flow cytometry (CyFlow Cube6, Sysmex) and both cell suspensions were diluted to equal cell concentrations.

Two approaches were tested for the generation of necromass: (i) cells were lysed by applying pressure to cell suspension using a French press, or (ii) cell suspensions underwent three cycles of autoclaving (121°C, 20 min) following Dong et al. (54). Afterward, 100 μL of the generated necromass were spread on sterile S2P-plates and incubated for 10 days, to guarantee that no viable cells were left in the necromass solutions. Because remaining growth was observed following the French press approach, only necromass produced by autoclaving was used for the mesocosm experiments. The necromass solutions were stored at −20°C until further usage.

### Study site and groundwater sampling.

The Hainich CZE is in the northwest of Thuringia (Germany) and facilitated access to two superimposed aquifer assemblages via multiple groundwater wells (30). In October 2018, groundwater was pumped from one well (H41) using a submersible motor pump (MP1, Grundfos, Denmark) as previously described (55). Groundwater was filled into sterile 10 L glass bottles, closed with Teflon lids (DWK Life Science), and cooled on blue ice until further processing.

### Incubation setup, DNA, and protein extraction.

In total, 21 10-liter bottles were filled with 9 liters of groundwater directly after sampling. While three bottles were used to determine the initial microbial community (day 0), the remaining 18 bottles were supplemented with ^12^C or ^13^C labeled necromass equivalent to 2 × 10^8^ cells/liter. While the average abundances of bacteria at this sampling site range between 1 × 10^7^ and 6 × 10^7^ cells/liter (27), a large amount of biomass added corresponds to biomass that can be introduced to groundwater via seepage (11). The water within the bottles without added necromass was directly filtered on 0.1 μm polyethersulfone (PES) filters (Supor, PALL Corporation) for biomass collection. All other mesocosms were incubated at 15°C in the dark for the entire course of the experiment. Oxygen concentrations were monitored daily using an FDO 925 sensor (WTW; Table S1). On days 2, 4, and 8 the entire water of three ^12^C, as well as three ^13^C-mesocosms was filtered on 0.1 μm PES filters (Supor, PALL Corporation) for biomass collection. All filters were directly stored at −80°C until further processing.

DNA and proteins were extracted from the filters as previously described (56) with slight modifications. No beads were added to the lysis solution. Instead, lysis was performed during a 2 h of incubation at 60°C. DNA pellets were resuspended in 50 μL nuclease-free water and stored at −20°C until further processing.

### Sequencing, sequence analysis, and quantitative PCR.

To test whether the necromass still contained amplifiable DNA and to assess the bacterial diversity in each mesocosm as well as the original groundwater at day 0, high-throughput sequencing of 16S rRNA genes using the primer combination 341F (5′-CCTACGGGNGGCWGCAG-3′) and 785R (5′-GACTACHVGGGTATCTAATCC-3′) (57, 58) was performed on the necromass suspension as well as DNA extracted from the original groundwater and mesocosms. No amplicon could be obtained from the necromass. Sequencing was carried out using the Illumina MiSeq platform and V3 Chemistry (Illumina). Adapter sequences were removed from the raw sequences using cutadapt (59) and the remaining sequences were analyzed using Mothur v.1.39.1 (60), following the Mothur MiSeq SOP (61) along with the SILVA bacteria reference alignment v132.

Abundances of bacterial 16S rRNA and eukaryotic 18S rRNA genes were obtained via quantitative PCR (qPCR) using the Brilliant II SYBR Green qPCR Mastermix (Agilent Technologies) on an Mx3000P instrument (Agilent Technologies). Bacterial 16S rRNA genes were amplified using the primer combination Bac8Fmod (5′-AGAGTTTGATYMTGGCTCAG-3′) and Bac338Rabc (5′-GCW GCC WCC CGT AGG WGT-3′) (62, 63). For eukaryotic 18S rRNA genes, the primers Euk-A7-F (5′-AACCTGGTTGATCCTGCCAGT-3′) and Euk-516R (5′-ACCAGACTTGCCCTCC-3′) (64, 65) were used.

### Proteomics.

The obtained protein extracts underwent an SDS polyacrylamide gel electrophoresis as well as a subsequent in-gel tryptic digestion. Peptides were purified and concentrated following Taubert et al. (56). Subsequently, the peptides were resuspended in 0.1% formic acid (vol/vol) for the following LC-MS/MS analysis on a Q Exactive HF instrument (Thermo Fisher Scientific) equipped with a TriVersa NanoMate source (Advion Ltd.) in LC chip coupling mode. A volume of 5 μL of the peptide lysates was separated via an Ultimate 3000 nanoRSLC-system (Dionex/Thermo Fisher Scientific).

Proteome Discoverer (v1.4.0288, Thermo Scientific) was used to identify the proteins and the acquired MS/MS spectra were searched against a custom database created from Uniprot based on taxonomic information of the community composition within the incubations based on 16S rRNA gene amplicon sequencing using the SequestHT algorithm (66). Trypsin was picked as the cleavage enzyme, allowing a maximum of two missed cleavages. A precursor mass tolerance (in MS) of 10 ppm and a fragment mass tolerance (in MS/MS) of 0.05 Da were applied. Carbamidomethylation of cysteine was considered fixed and oxidation of methionine was set as a dynamic modification. The peptide spectrum matches were validated using Percolator (v2.04) with a false discovery rate (FDR) < 1% and quality filtered for XCorr ≥2.25 (for charge state +2) and ≥2.5 (for charge state +3). Identified peptides were grouped using the strict parsimony principle (67).

The taxonomic classification of peptides was based on the lowest common ancestor method in UniPept (68). The functional classification of peptides was based on KEGG orthology assignments to the corresponding protein sequences obtained with kofamscan (69). Only hits scoring over 80% of the protein-specific thresholds, or over 100 for proteins without a threshold, were considered for functional analysis. The identification of ^13^C-labeled peptides as well as the quantification of incorporation of ^13^C was done by comparing measured and expected isotopologue patterns, chromatographic retention times, and fragmentation patterns (70, 71).

### Data analysis.

Analysis of the data was conducted in R (72) within RStudio (73), using the packages vegan (74), tidyr (75), and forcats (76). Visualization in R was performed with the packages ggplot2 (77) and ggrepel (78).

### DNA-SIP.

DNA from the final time point was used for DNA stable isotope probing (SIP) following Taubert et al. (79). In brief, 2 to 3 μg of DNA was added to a mixture of gradient buffer (0.1 M Tris, 0.1 M KCl, 1 mM EDTA; pH 8) and 7.2 M CsCl and the final density was adjusted to 1.725 g/mL. Ultracentrifugation in a Sorvall Discovery 90SE (Hitachi) and an NVT 90 rotor was carried out for 70h at 20°C and 44,100 rpm. Subsequently, the mixture was separated into 13 fractions, fraction densities were calculated based on refractive indices (AR200 refractometer, Reichert technologies, Buffalo, USA) and DNA was precipitated and washed following Taubert et al. (79). The heavy and light DNA fractions of the ^13^C incubations, as well as the corresponding fractions of the ^12^C incubations, were selected for 16S rRNA gene amplicon sequencing as well as for 18S rRNA gene qPCR to determine the abundances of eukaryotes based on their density (ρ_heavy_=1.73, ρ_light_= 1.69) following (32).

### Data availability.

The reference database used for the proteomics data analysis and all sequence data are publicly available via the Open Science Framework (osf.io) and can be accessed via https://osf.io/59y3w/?view_only=3d54f4d84a8e4ba8a8d61a8f50bba55f. Additionally, all sequence data were deposited at the European Nucleotide Archive (ENA) and can be accessed via the project PRJEB46919. The mass spectrometry proteomics data have been deposited to the ProteomeXchange Consortium via the PRIDE (80) partner repository with the data set identifier PXD031173.
